# Probing the Selectivity of Monoamine Transporter Substrates by Means of Molecular Modeling

**DOI:** 10.1002/minf.201300013

**Published:** 2013-06-12

**Authors:** Amir Seddik, Marion Holy, René Weissensteiner, Barbara Zdrazil, Harald H Sitte, Gerhard F Ecker

**Affiliations:** [a] University of Vienna, Department of Medicinal Chemistry, Pharmacoinformatics Research GroupVienna, Austria; [b] Medical University Vienna, Center for Physiology and Pharmacology, Institute of PharmacologyVienna, Austria

**Keywords:** Serotonin transporter (SERT), Dopamine transporter (DAT), Substrate selectivity, Fenfluramine, Docking, Common scaffold clustering

The structurally similar serotonin and dopamine transporter (resp. SERT and DAT) play an important role in neuronal transmission. Although the concept of their function, i.e. the re-uptake of neurotransmitters from the synaptic cleft, has been extensively studied,^1^–^4^ the exact mechanism for their substrate selectivity is still unknown. Phenylethylamines (PEAs) are ligands of SERT and DAT and many induce reverse transport (efflux) of the protein’s natural substrate (the neurotransmitters 5-hydroxytryptamine and dopamine) in varying degrees and with different kinetics.^2^,^5^–^7^ Thus, studying the interplay of bioactivity values and certain structural features of selected PEAs can lead to new insights about monoamine transporter selectivity. The broadest SAR data currently available for PEAs and their interaction with SERT and DAT has been measured in rat synaptosomes by Baumann and colleagues.^8^,^9^

Thus, we used this data set to figure out important features which contribute towards selectivity and to guide the selection of a probe compound for subsequent structure-based studies. Consequently, p*EC*_50_ values of 28 compounds for SERT and DAT (Table 1) were plotted against each other, providing a clear picture of the PEA′s selectivity profile (Figure 1). Out of this, a couple of detailed SARs can be drawn:

- Chirality of the α-methylene atom of amphetamines does not influence SERT/DAT selectivity.
- The (*S*)-enantiomer is the most active in both transporters.
- DAT selective substrates seem smaller in size and therefore, their conformational flexibility in the binding pocket is expected to be relatively high and interactions with the target less defined.
- *N*-Methyl substitution slightly increases activity in SERT (compare compounds **4**, **8**, **20** and **21**), and is somewhat unchanged in DAT (compare compounds **16**, **17**, **18** and **19**). The only exception is for the naphtylisopropylamine (NIPA, **23**) which is not selective for both transporters and shows a slight decrease in SERT activity (**24**).
- *N*-Ethyl substitution is generally more favorable in SERT as compared to methyl substitution or no substitution, while it decreases activity in DAT (see compounds **19**, **22** and **25**).
- *para*-Chlorine, *meta*-CF_3_ or *meta*-methyl substitution dramatically increases SERT affinity (compare **9**, **11**, **12**, **4**, **17**).
- β-Hydroxyl substitution (R_4_, Table 1) decreases affinity in both SERT and DAT (compare **1**, **3**, **5**, **7**).
- *para*-Methyl substitution increases SERT affinity and slightly decreases DAT affinity (compare **4**, **10**, **26**, **27**).

**Table 1:** Monoamine transporter substrate structure-activity relationships

Cpd	Name	R_1_	R_2_	R_3_	R_4_	R_5_	R_6_	p*EC*_50_ rDAT	p*EC*_50_ rSERT
	**Phenylethylamines**								
	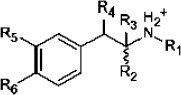								
1	Dopamine	H	H	H	H	OH	OH	7.1	5.0
2	Tyramine	H	H	H	H	H	OH	6.9	5.6
3	Norepinephrine	H	H	H	*(S)*-OH	OH	OH	6.1	5.0
4	(*S*)-Amphetamine	H	Me	H	H	H	H	7.6	5.6
5	(*R*)*-*Ephedrine	Me	Me	H	(*S*)-OH	OH	OH	5.9	5.0
6	HMA ^[a]^	H	Me	H	H	H	OH	5.5	6.1
7	(*R*)-Methamphetamine	Me	Me	H	H	H	H	6.4	5.3
8	(*S*)-Methamphetamine							7.6	6.1
9	*m*-Methylamphetamine ^[a]^	H	Me	H	H	Me	H	7.5	6.7
10	*p*-Methylamphetamine ^[a]^	H	Me	H	H	H	Me	7.5	6.7
11	Phentermine	H	Me	Me	H	H	H	6.6	5.5
12	Chlorphentermine	H	Me	Me	H	H	Cl	5.6	7.5
13	*m*-Fluoroamphetamine ^[a]^	H	Me	H	H	F	H	7.6	5.7
14	*p*-Fluoroamphetamine ^[a]^	H	Me	H	H	H	F	7.3	6.0
15	HMMA ^[a]^	Me	Me	H	H	MeO	OH	5.5	6.2
16	(*R*)-Norfenfluramine	H	Me	H	H	CF_3_	H	5.0	6.5
17	(*S*)-Norfenfluramine							6.0	7.2
18	(*R*)-Fenfluramine	Et	Me	H	H	CF_3_	H	5.0	6.8
19	(*S*)-Fenfluramine							5.0	7.3
		R_1_	**3,4-Methylenedioxyamphetamines**						
20	MDA ^[a]^	H	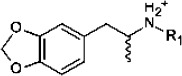					7.0	7.0
21	(*S*)-MDMA	Me						7.3	7.3
22	(*S*)-MDEA	Et						6.3	7.3
		R_1_	**Naphtylisopropylamines**						
23	NIPA ^[a]^	H	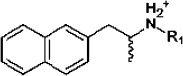					7.8	8.4
24	(*S*)-*N*-Methyl-NIPA	Me						8.0	7.9
25	(*S*)-*N*-Ethyl-NIPA	Et						7.3	7.9
			**Cathinones**				R_6_		
26	Methcathinone ^[a]^		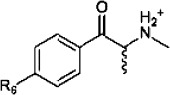				H	7.7	5.4
27	Mephedrone ^[a]^						Me	7.3	6.9
			**Other**						
28	PAL-738		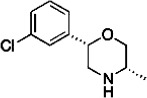					7.2	7.6

[a] Chiral amphetimes without designated configuration represent the racemic mixture; H: hydrogen, Me: methyl, Et: ethyl, OH: hydroxy, MeO: methoxy, CF_3_: trifluoromethyl

**Figure 1:**
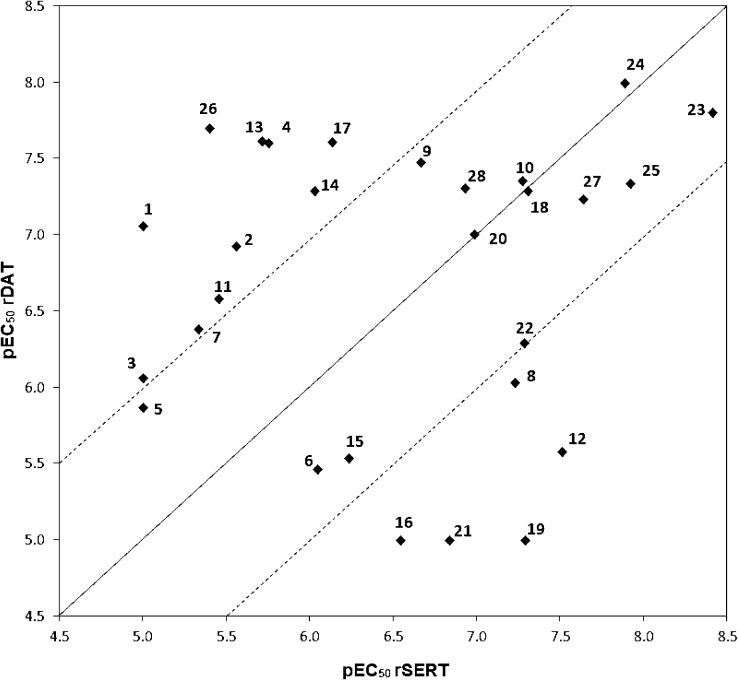
Selectivity plot with numbers corresponding to Table 1. Compounds with similar SERT/DAT affinity are located around the middle diagonal line, while compounds in the upper left corner and lower right corner are DAT and SERT-selective, respectively.

The highest SERT/DAT selectivity is shown by (*S*)*-*fenfluramine (SFF) and because of its relatively large size, docking studies with this ligand are expected to result in a more restricted amount of poses as compared to the smaller analogs. Subsequently, we used SFF as a probe compound in order to study the molecular basis of the high affinity and selectivity of this compound towards SERT by means of a structure-based approach. Conveniently, sequence identity between the human and rat transporters is very high (92 % with SERT; 93 % with DAT), and local alignment of the primary substrate binding site (S1^4^) shows even 100 % sequence identity between both species.^10^ Thus, in order to build upon our already established protein homology models for human SERT^11^, we switched to human proteins for subsequent studies.

To show that data derived from rat transporters indeed can be transferred to the human transporters, we confirmed the high selectivity of SFF for SERT employing an uptake inhibition assay on HEK cells expressing human SERT and DAT (*IC*_50_=5.89 µM in SERT and 118 µM in DAT, see Figure 2).

**Figure 2:**
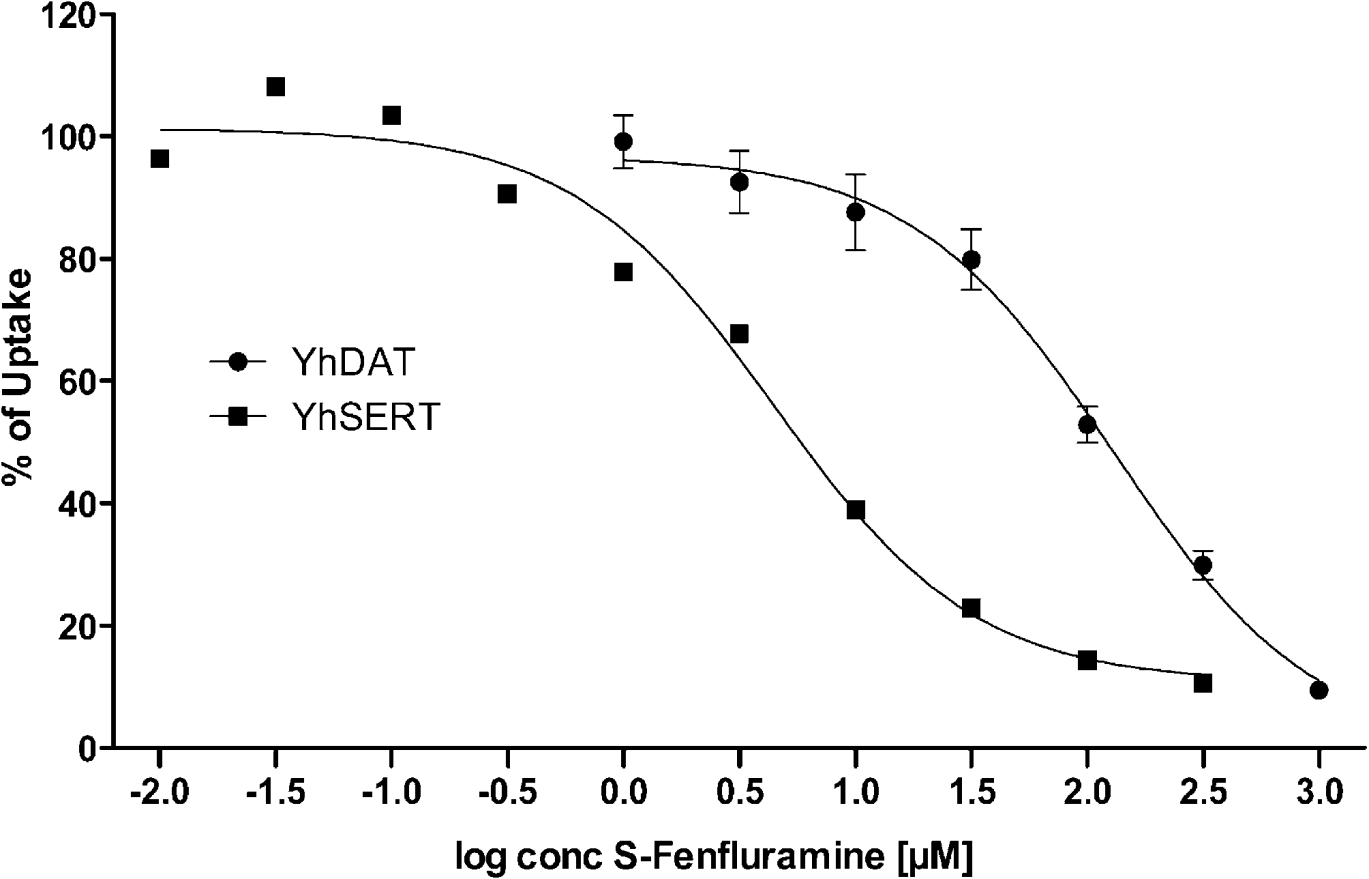
Uptake inhibition by (*S*)-fenfluramine in HEK293 cells stably expressing YFP-tagged DAT and SERT. Uptake was inhibited by increasing concentrations of fenfluramine as indicated. The concentration of tritiated substrates was 0.15 µM in the case of [^3^H]5HT while 0.1 µM was used for [^3^H]DA. Data are shown as means±SEM of three (DAT) or four (SERT) independent experiments carried out in triplicate.

Docking of a set of diverse high-affinity SERT substrates (see Methods) into a homology model of hSERT followed by common scaffold clustering revealed a binding mode for SFF which is in accordance to previously published studies.^12^,^13^ In addition, SFF was docked into an analogously constructed homology model of hDAT. Results showed that this ligand fits nicely into the S1 site, meaning that steric hindrance caused by the trifluoromethyl or *N*-ethyl group could not serve as an explanation for its low DAT affinity (see Figure 3). In addition, scoring functions could not show a preference of SFF for SERT or DAT (see Table 2) and hence are not able to capture the activity determining factors. Since SFF′s trifluoromethyl moiety seems to be driving the selectivity, we further analysed the pocket between the TM3 and TM8 helical domains where this moiety is located: local alignment of SERT and DAT showed that five of the seven residues within this pocket are different. In general, the SERT pocket has more lipophilic side chains in its binding site, except for Thr439 in SERT which is more hydrophilic than the corresponding Ala423 in DAT (see Table 3).

**Figure 3:**
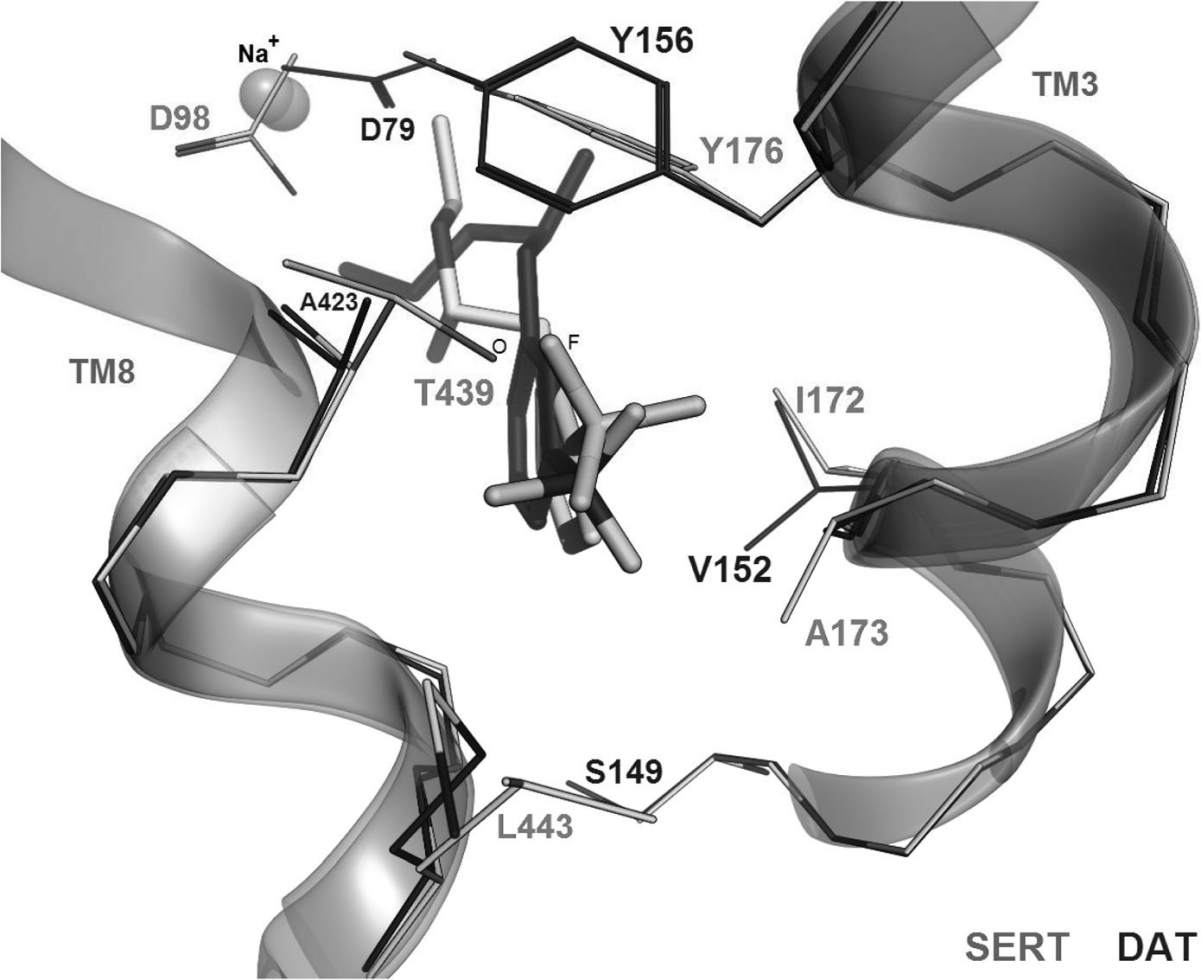
Overlay of the selected fenfluramine (SFF) poses in the substrate binding site of hSERT and hDAT with a T439(O)-F(SFF) distance of 3.5 Å.

**Table 2:** Average scoring values after docking and evaluation of *(S)*-fenfluramine in the substrate binding site of homology models of SERT and DAT

	SERT	DAT
X-score (−*K*_D_ in kcal/mol)	6.5±0.1	6.4±0.1
DSX	−85±9	−85±12
London dG	−12.4±1.5	−12.7±0.7
*N poses*	14	9

**Table 3:** Local alignment of the helical domains TM3 and TM8 of hSERT and hDAT showing more lipophilic side chains in SERT, except that for Thr439

SERT	A169	I172	A173	Y176	T439	G442	L443
DAT	S149	V152	G153	Y156	A423	G426	M427

This indicates a potential role of the CF_3_ group and Thr439 for SERT selectivity. Furthermore, as shown in Table 1, (*S*)*-*amphetamine and (*S*)*-*norfenfluramine only have a trifluoromethyl moiety dissimilar, and their *K*_i_ values for rSERT are 3830 nM and 214 nM, respectively.^5^,^8^ Since the ratio of these values should be similar to the *K*_D_ ratio (and since K_i_ is comparable to *K*_D_^14^), the binding free energy formula can be applied:


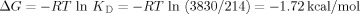


with *T*=298.15 K^5^

and


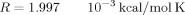


Hence, from a ligand-based point of view, a more favorable binding energy of about 1.72 kcal/mol is calculated for (*S*)-norfenfluramine. Considering the inhibitory values of (*S*)*-*fenfluramine from our human DAT and SERT uptake inhibition assay, we obtain a binding free energy difference of about 1.75 kcal/mol:^15^


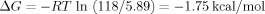


with *T*=293.15 K

Both calculated energy values are close to each other, strengthening the evidence that the trifluoromethyl group is responsible for SERT/DAT selectivity and high SERT affinity. Moreover, these values are relatively close to the Δ*G* value of a sp^3^-fluorine hydrogen bond (−2.38 kcal/mol).^16^ It is thus tempting to speculate that an interaction of Thr439 with the CF_3_ group triggers both affinity and selectivity of SFF in SERT. In addition, lipophilic dispersion forces with the SERT specific side chains (Ala169, Ile172, Ala173) that surround the trifluoromethyl moiety might contribute. Further evidence for the potential role of the lipophilicity of this pocket can be deduced from the increase in activity of the more lipophilic *meta*-methyl-substituted compound **9** and a decrease in activity of the hydrophilic *meta*-hydroxy-substituted dopamine (**1**) and norepinephrine (**3**) in this protein. Finally, when comparing phentermine and chlorphentermine, the halide increases the SERT affinity 13 900/338=41 times,^5^ which corresponds to more favorable energy of about 2.21 kcal/mol. Whether this can be ascribed to an interaction between the chlorine and Thr439, or simply to lipophilic contributions, is a point of discussion.

With this study we have shown that combining ligand- and structure based studies are a powerful tool to probe substrate selectivity of monoamine transporters leading to preliminary evidence for the potential role of halogen atoms and Thr439 in SERT. Synthesis of additional PEAs combined with biochemical studies in both wild type and T439A mutants are obvious further steps towards this direction.

## Experimental

*Materials and Methods*. Dulbecco’s modified Eagle’s medium (DMEM) and trypsin were purchased from PAA Laboratories GmbH (Pasching, Austria). Fetal calf serum was purchased from Invitrogen. [^3^H]5HT ([^3^H]5-hydroxytryptamine; serotonin; 28.3Ci/mmol) and [^3^H]DA (dopamine; 35 Ci/mmol) were purchased from PerkinElmer, Boston, MA, USA. Serotonin (5HT), dopamine (DA) and SFF were purchased from Sigma.

*Uptake Inhibition Assays.* The generation of HEK293 cell lines expressing Yellow Fluorescent Protein (YFP)-tagged hSERT and hDAT is described earlier (Sucic et al. 15). HEK293 cells stably expressing either SERT or DAT were seeded onto poly-d-lysine-coated 48-well plates (0.5×10^5^ cells/well), 24 hours prior to the experiment. For inhibition experiments, the specific activity of the tritiated substrate was kept constant: [^3^H]DA: 0.1 µM, [^3^H]5HT: 0.15 µM. Assay conditions were as outlined;^15^ in brief: the cells were washed thrice with Krebs-Ringer-HEPES buffer (KHB; composition: 25 mM HEPES.NaOH, pH 7.4, 120 mM NaCl, 5 mM KCl, 1.2 mM CaCl_2_, and 1.2 mm MgSO_4_ supplemented with 5 mM d-glucose). Then, the diluted reference and sample compounds were added and incubated for 5 minutes to allow for equilibration with the transporters. Subsequently, the tritiated substrates were added and the reaction was stopped after 5 minutes. Cells were lysed with SDS 1 % and counted in a beta-counter (Packard instruments). All determinations have been performed in triplicate.

*Homology Modeling.* Models of the human SERT and DAT were created as described by Sarker et al.^11^ using LeuT_Aa_ in the occluded conformation (PDB id 2A65, 1.65 Å)^17^ as template. The highest DOPE scored structure was energy minimized in the AMBER99 forcefield and underwent a quality check using the QMEAN server. The binding site was defined using the Site Finder tool of Molecular Operating Environment.

*Docking.* Nine structurally diverse PEAs with high SERT affinity (**10**, **12, 19, 21, 22, 25, 27, 28, 29**) were docked into the S1 of SERT using CCDC GOLD 5.0.1. In case of an amphetamine, only the (*S*) enantiomer was docked. SFF was docked into the DAT S1 alone because its low affinity could cause a distinct conformation in the binding site. One hundred poses per ligand were generated and the ligand and residue’s side chains within a 6Å radius were set as freely flexible (10 degree bins). Poses not comprising a required ionic interaction with the D79 (DAT) and D98 (SERT) side chain^18^ were discarded, leading to 45 SFF-SERT and 65 SFF-DAT complexes. The ligand and surrounding atoms within a 8 Å radius were energy minimized in the Merck Molecular Forcefield (MMFF94x). Common scaffold clustering was applied on the SERT complexes, whereby the PEA scaffold was extracted from each complex and an RMSD matrix based on its heavy atoms was calculated.^19^ Agglomerative hierarchical clustering, using XLStat (complete linkage, cutoff level 3), led to 13 clusters. Those clusters not containing all ligands were discarded, leading to 7 clusters comprising 41 SFF poses. From the top 10 scored poses of X-Score^20^ and DSX scoring function,^21^ one consensus pose was found and from the 14 complexes of the cluster containing this pose, the average rescoring values were calculated. For the DAT poses, 11 clusters were obtained of which two had a similar ligand orientation (the aromatic ring in the same position) as in the consensus SERT pose. From these two clusters (9 poses), the average scores were calculated (Table 2).
